# The impact of COVID-19 on longevity trends and disparities among Native Americans and Whites in the Four Corners States

**DOI:** 10.1371/journal.pone.0347924

**Published:** 2026-05-18

**Authors:** Ashley M. Lenarz, Hyojun Park, Max T. Roberts, Anwesha Pan, Erin Trouth Hofmann, Eric N. Reither

**Affiliations:** 1 Yun Kim Population Research Laboratory, School of Social Sciences, Utah State University, Logan, Utah, United States of America; 2 Independent Researcher, Lacey, Washington, United States of America; UCSF: University of California San Francisco, UNITED STATES OF AMERICA

## Abstract

Prior research has shown suboptimal health and longevity among Native Americans in the Four Corners region of the United States, which encompasses Arizona, New Mexico, Colorado, and Utah. Our study (1) investigates how life expectancy trends and disparities changed among non-Hispanic Native Americans and Whites in the Four Corners States (FCS) during the COVID-19 pandemic and (2) examines the extent to which longevity changes are directly attributable to COVID-19, relative to other causes of death. Data sources include mortality data from the National Center for Health Statistics and population data from the U.S. Census Bureau. Life expectancy at birth for four race-sex groups in the FCS (Native American and White females and males) was calculated using abridged life table procedures, both pre-pandemic (2018−19) and peak pandemic (2020−22). Gaps in life expectancy between groups (and changes within groups) were decomposed into multiple causes of death to determine which causes contributed most to life expectancy gaps and changes across time periods. Life expectancy declined in the FCS over the study period; whereas Native American male and female longevity decreased by 7.33 years and 6.76 years, respectively, White male and female longevity decreased by 2.11 years and 1.72 years, respectively. Results indicate that the peak pandemic life expectancy gap between Native Americans and Whites widened by over 5 years, regardless of sex. Although COVID-19 was the single largest contributor to longevity changes within and between groups, causes of death related to drug and alcohol use also made notable contributions, especially among Native Americans. Restoring longevity to pre-pandemic levels in the FCS will require improved management of COVID-19 as well as heightened attention to the deleterious role of substance use in indigenous communities.

## Introduction

While no race or ethnic group in the United States (US) was unaffected by the COVID-19 pandemic, Native Americans were disproportionately impacted. According to the US Census Bureau, American Indians and Alaskan Natives (AIAN) experienced a mortality increase of 36.7% in 2020, the largest increase in any US racial/ethnic group [1]. Indigenous people in the Four Corners states (FCS), which encompasses Arizona, New Mexico, Colorado, and Utah, were especially vulnerable to the adverse impacts of COVID-19, including severe illness and premature mortality [2,3]. To illustrate, relative to non-Hispanic Whites, COVID-19 standardized mortality ratios among Native Americans in Arizona (8.3) and New Mexico (9.9) were much higher than most other states, such as California (1.7), Texas (0.9), Oklahoma (2.3), Minnesota (2.7), and Michigan (1.5) [2].

Prior research in the FCS has established a persistent life expectancy disadvantage among the non-Hispanic AIAN population. For example, a pre-pandemic study in the FCS found notable differences in life expectancy between non-Hispanic AIAN and White populations, both for males (4.92-year gap) and females (2.06-year gap) [4]. A more recent study examining US AIAN longevity during the pandemic found that, relative to 2019, life expectancy decreased by 4.5 years in 2020 and 6.4 years in 2021 [5]. Moreover, US AIAN life expectancy in 2022 was still four years lower than in 2019 [6]. Research suggests that an increase in non-COVID deaths such as drug overdose, chronic liver disease, diabetes, and heart disease contributed to the stagnation of US AIAN life expectancy in 2022 [6]. To date, however, no study has examined how COVID-19 impacted longevity trends and disparities among Native Americans and Whites in the FCS, relative to other causes of death such as drug and alcohol use. Consequently, the overarching objective of this study is to investigate how the COVID-19 pandemic impacted longevity trends and disparities among indigenous people in the FCS.

### FCS Tribes

According to the 2010 Census, over 500,000 individuals in the FCS identified as single race AIAN [7]. The FCS has numerous tribal lands and reservations including Navajo Nation, which is the largest reservation in the United States [8]. New Mexico has 23 federally-recognized tribes; Arizona has 22, Utah has eight, and Colorado has two [9–12]. In addition to Navajo, the FCS is home to Apache, Goshute, Hopi, Hualapai, Paiute, Pascua Yaqui, Pueblo, Quechua, Shoshone, Tohono O’odham Ute, Yavapai, and Zuni tribes, among others [9–12].

### COVID-19 risk factors

Relative to non-Hispanic Whites, Native Americans have higher rates of chronic disease and poverty with lower rates of health insurance coverage and health literacy, which increases their risk for severe COVID-19 and limits their ability to prevent transmission and access medical care [2,3,13–15]. Indigenous people also have higher rates of asthma, COPD, heart and kidney disease, obesity, and diabetes [2,16]. Relative to other racial/ethnic groups, Native Americans are more likely to live below the poverty line, work in a frontline job (i.e., non-remote jobs that require frequent proximity to other people), or be unemployed [2,3,15]. AIAN individuals have the lowest rates of health insurance coverage among all racial/ethnic groups in the US [2,3,13]. For example, in 2021, the uninsured rate for non-Hispanic AIAN was 18.8%, compared to just 5.7% for non-Hispanic Whites [13]. Furthermore, private health insurance coverage was significantly lower among non-Hispanic AIAN individuals (43.1%) than among non-Hispanic Whites (74.2%) [13]. Additionally, historical trauma and discrimination have led to mistrust of the health care system and COVID-19 vaccination hesitancy among indigenous groups, creating additional barriers to care and prevention [17,18]. All these factors have likely contributed to a disproportionate number of COVID-19 cases and deaths in AIAN communities.

Prior research has shown that residents of Navajo Nation experience some of the most acute risks to health and longevity in the US, even relative to other indigenous groups [3,19–21]. Navajo Nation has very high percentages of residents in poverty (40.2%), adults without a high school diploma (26.9%), overcrowded housing conditions (17.4%), households without a vehicle (14.4%), and households with incomplete plumbing (18%) [2,3,21]. Additionally, a relatively large number of Pueblo and Navajo households do not speak English, which can make it difficult to secure resources such as employment and adequate healthcare [21]. Moreover, many reservations in the FCS are highly rural and lack advanced medical facilities capable of treating severe cases of COVID-19 [19,20].

The two primary aims of this study are to (1) investigate how the COVID-19 pandemic impacted life expectancy trends among non-Hispanic AIAN and Whites in the FCS, and (2) understand the extent to which changes in longevity and longevity disparities in the FCS are directly attributable to COVID-19 versus other causes of death. This study will decompose life expectancy changes *within* (and gaps *between*) AIAN and White populations in the FCS, allowing us to determine which causes of death contributed most to these changes and gaps during the COVID-19 pandemic. As noted, while some prior studies have examined how COVID-19 impacted longevity among Native Americans, none have employed decomposition methods to disentangle COVID-19 from other causes of death among AIAN and Whites in the FCS region.

## Methods

### Data

To assess longevity changes in the FCS over the period 2018–2022, single-race population data from the US Census Bureau for Arizona, Colorado, New Mexico, and Utah were utilized [22]. In addition, restricted-use mortality data (2018–2022) sourced from the US National Center for Health Statistics (NCHS) were included in our investigation [23].

### Measures

Measures in our data include sex, age, race/ethnicity, and cause-of-death. Age at death was organized into 19 categories (less than 1 year old, 1–4 years old, a series of five-year age groups from 5–9 to 80–84, and 85 and above). The measurement for race was based on six revised single-race categories, which includes White, Black or African American, Asian, AIAN, Native Hawaiian or Other Pacific Islander, and more than one race [24]. Analyses included single race non-Hispanic AIAN (hereafter NH AIAN) and non-Hispanic White (hereafter NH White) populations in the FCS [25]. A small number of individuals identified as belonging to “multiple races” was excluded from the analyses. The analyses also excluded the Hispanic population because this group often exhibits socioeconomic, cultural, behavioral, and health profiles that differ from non-Hispanic populations [26].

The 10th revision of the International Classification of Diseases (ICD-10) was utilized to identify the 39 leading causes of death in the US [27]. These categories are mutually exclusive and exhaustive, encompassing a wide range of medical conditions and external causes, including specific types of heart disease and cancer, chronic lower respiratory diseases, cerebrovascular disease, Alzheimer’s disease, diabetes, influenza and pneumonia, and nephritis, among others [27]. Analyses also incorporated alcohol-related causes of death as defined by the Alcohol-Related Disease Impact (ARDI) classification scheme, by isolating different ARDIs and then subtracting them from the 39 leading causes of death [28]. ARDIs were classified into chronic or acute conditions that are either 100% attributable to alcohol (e.g., acute alcohol poisoning) or have alcohol-attributable fractions (AAF) that are directly related to alcohol use (e.g., fatal motor vehicle crashes involving alcohol use) [28]. A detailed description and the complete list of ICD-10 codes used in the ARDI application are available elsewhere [28]. S1 Table provides a list of ICD-10 codes for the 39 causes of death and ARDI.

### Analysis

The first step was to construct a series of sex-specific period life tables for NH AIAN and NH White populations, spanning the period 2018–2022. Using death records from the NCHS and single-race population data from the U.S. Census Bureau, sex- and age-specific death rates (ASDR) were calculated for both populations. Sex-specific ASDR were used to estimate life expectancies at birth for NH AIAN and NH White populations in the FCS across the study period, following standard procedures for creating abridged life tables [29].

The second step of the analysis involved decomposing life expectancy gaps into age- and cause-of-death components, using the approach proposed by Arriaga [29,30]. Data for decomposition analyses were combined into two- or three-year aggregates of deaths and population data (i.e., pre-pandemic, 2018−19 and peak pandemic, 2020−22), similar to approaches used in previous research [4]. The total life expectancy gap was decomposed into years according to the relative contributions of each age group, followed by the specific causes of death within each age group. Age- and cause-specific decompositions were applied to the life expectancy differences for males and females 1) between NH AIAN and NH White populations at two different time points (i.e., 2018−19 and 2020−22) and 2) within these two populations before and after COVID-19.

The final step was to identify causes of death that were particularly relevant to our selected populations in the FCS. Among the 39 leading causes of death and ARDI we examined, select causes were presented in our final report if they contributed at least 0.20 years to 1) the life expectancy gap between NH AIAN and NH White populations for either males or females in at least one time period, or 2) changes in life expectancy for at least one race/sex group that occurred after the onset of COVID-19. For between-group differences, causes of death that met these criteria included COVID-19, diabetes mellitus, heart disease, malignant neoplasms (i.e., various cancers), symptoms and abnormal clinical/laboratory findings not elsewhere classified (excluding sudden infant death syndrome), influenza and pneumonia, nephritis/nephrotic syndrome/nephrosis, Alzheimer’s disease, chronic lower respiratory diseases, other external causes and unspecified accidents, alcohol abuse and poisoning, alcoholic liver disease, alcohol-related liver cirrhosis, alcohol-related vehicle crashes, alcohol-related homicide, non-alcohol poisoning, and alcohol-related suicide. For within-group changes, the only causes of death that met our inclusion criteria were COVID-19, heart disease, alcoholic liver disease, alcohol-related liver cirrhosis, and non-alcohol poisoning. In both analyses, we included a residual category to account for all other causes of death that did not meet our inclusion criteria.

In line with our research objectives, the results were presented in three parts: first, an overview of life expectancy trends; second, a detailed analysis of the *gaps* in life expectancy between race/sex groups; and third, an examination of life expectancy *changes* within each race/sex group. Analyses were conducted using SAS version 9.4 (Cary, NC), R (R Core Team), and MS Excel [30]. Restricted data for the analyses were obtained with permission from the NCHS (Contract number: DVS2024−0259). Our university’s institutional review board considered this study exempt from review (protocol number: 14619).

## Results

**Fig 1:**
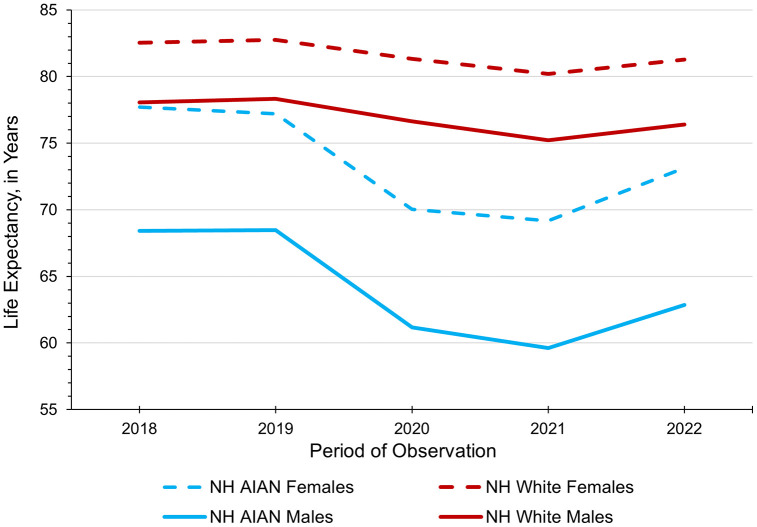
Life expectancy among NH AIAN and NH Whites in the Four Corners States, 2018-2022. Abbreviations. NH (Non-Hispanic); AIAN (American Indian or Alaska Native).

Fig 1 illustrates FCS trends in life expectancy at birth by sex and race/ethnicity from 2018 to 2022. In 2018, life expectancy was 82.56 years for NH White females, 78.06 years for NH White males, 77.70 years for NH AIAN females, and 68.41 years for NH AIAN males. These life expectancies were relatively stable in 2019 but dropped substantially in the first year of the COVID-19 pandemic (i.e., 2020), especially among NH AIAN people. In 2021, longevity reached its lowest level among all four groups, falling to 80.19 years for NH White females, 75.23 years for NH-White males, 69.18 years for NH AIAN females, and 59.62 for NH AIAN males. In 2022, life expectancy rebounded for each group but did not return to pre-pandemic levels. While the pandemic reduced life expectancy for both NH AIAN and NH White populations, it had an acute impact on NH AIAN males and females, who experienced sharp declines in longevity.

Table 1 summarizes the contribution of select causes of death to racial/ethnic life expectancy gaps, expressed as sex-specific differences between NH White and NH AIAN populations in each period of observation. During the COVID-19 pandemic, racial/ethnic longevity gaps grew by about five years, regardless of sex, increasing from 9.75 years in 2018−19 to 14.97 years in 2020−22 among males and from 5.20 years in 2018−19 to 10.24 years in 2020−22 among females. Before the pandemic, the 9.75-year life expectancy gap between NH AIAN and NH White males was largely attributable to alcoholic liver disease (1.25 years), alcohol-related motor vehicle traffic crashes (1.14 years), alcohol abuse and poisoning (1.13 years), all other external causes or unspecified accidents (0.99 years), and diabetes mellitus (0.89 years). During the pandemic, COVID-19 (3.20 years) and non-alcohol poisoning (0.85 years) emerged as key contributors to the 14.97-year gap among males, along with the aforementioned causes of death. Among females, the 5.20-year life expectancy gap prior to the pandemic was mainly attributable to alcoholic liver disease (1.18 years), diabetes mellitus (0.92 years), alcohol-related liver cirrhosis (0.57 years), alcohol-related motor vehicle traffic crashes (0.54 years), and alcohol abuse and poisoning (0.45 years). Similar to males, COVID-19 (3.15 years) and non-alcohol poisoning (0.38 years) emerged as important contributors to the 10.24-year longevity gap among females during the pandemic, in addition to the causes of death previously noted. Somewhat counterintuitively, several causes of death consistently favored NH AIAN over NH Whites, meaning they were less prevalent among AIAN, such as Alzheimer’s disease and chronic lower respiratory diseases among both males and females, and diseases of heart and malignant neoplasms among females.

**Table 1:** Contribution of select causes of death to life expectancy gaps between NH AIAN and NH White populations in the Four Corners States, by sex and period of observation.

Causes of Death^1^	Males		Females	
**2018-2019**	**2020-2022**	**2018-2019**	**2020-2022**
NH White Life expectancy	78.19	76.08	82.65	80.93
NH AIAN Life expectancy	68.44	61.11	77.45	70.69
**Life expectancy gap between NH AIAN and NH White populations**	**9.75**	**14.97**	**5.20**	**10.24**
COVID-19	–	3.20	–	3.15
Alcoholic liver disease	1.25	1.71	1.18	1.57
Alcohol abuse and poisoning	1.13	1.28	0.45	0.59
Alcohol-related motor vehicle traffic crashes	1.14	1.06	0.54	0.61
Diabetes mellitus	0.89	0.75	0.92	0.71
Alcohol-related liver cirrhosis	0.51	0.63	0.57	0.83
All other external causes or unspecified accidents	0.99	0.92	0.15	0.32
Alcohol-related homicide	0.67	0.74	0.22	0.18
Alcohol-related suicide	0.50	0.60	0.19	0.25
Poisoning (not alcohol)	0.06	0.85	0.08	0.38
Symptoms, signs and abnormal clinical and laboratory findings^2^	0.30	0.49	0.20	0.20
Influenza and pneumonia	0.31	0.28	0.32	0.22
Nephritis	0.21	0.16	0.33	0.19
Diseases of heart	0.19	0.39	−0.41	−0.17
Malignant neoplasms (cancers)	0.04	−0.02	−0.22	−0.08
Alzheimer’s disease	−0.14	−0.07	−0.44	−0.20
Chronic lower respiratory diseases	−0.30	−0.17	−0.45	−0.28
All other causes	1.98	2.18	1.58	1.76

*Note.* 1) Among the 39 leading causes of death and ARDI we examined, select causes of death were included in this table if they contributed at least 0.20 years to the life expectancy gap between NH AIAN and NH White populations for either males or females in at least one time period. Causes of death are listed in order of total contribution to life expectancy gaps. A residual category (all other causes) accounts for the total contribution of all other causes of death that did not meet our inclusion criteria. Whereas positive contributions indicate longevity disadvantages for the NH AIAN population relative to the NH White population, negative contributions indicate longevity advantages.

2) Further details regarding this category are available in the World Health Organization’s International Classification of Diseases, 10^th^ Revision, Chapter XVIII (https://icd.who.int/browse10/2019/en#/XVIII).

Abbreviations. NH (Non-Hispanic); AIAN (American Indian or Alaska Native); ARDI (Alcohol-Related Disease Impact)

**Fig 2:**
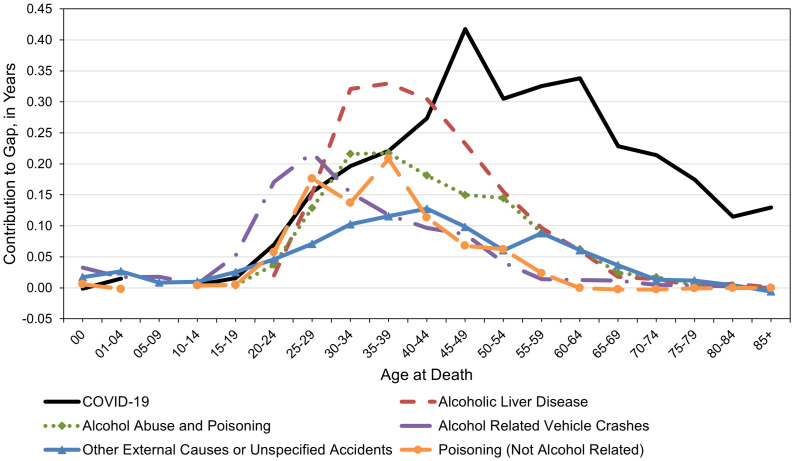
Age distribution of leading contributors to the male life expectancy gap between NH AIAN and NH White populations in the Four Corners States, 2020-2022. The longevity gap of 14.97 years in 2020−22 was decomposed into numerous causes of death at specific ages. The causes depicted here are the leading six contributors to this life expectancy gap. Abbreviations. NH (Non-Hispanic); AIAN (American Indian or Alaska Native).

**Fig 3:**
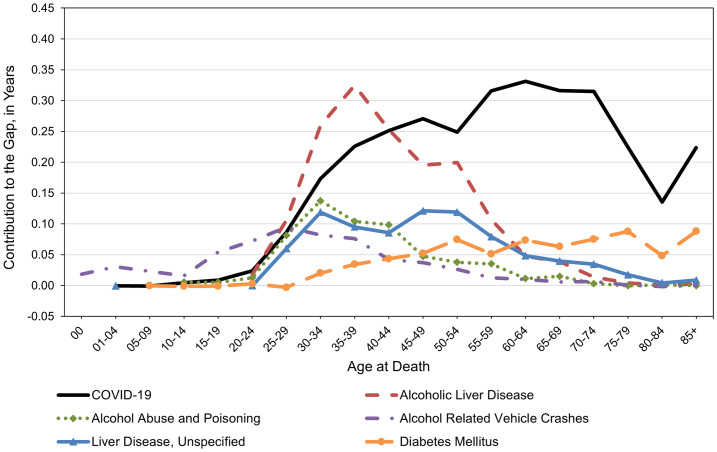
Age distribution of leading contributors to the female life expectancy gap between NH AIAN and NH White populations in the Four Corners States, 2020-2022. The longevity gap of 10.24 years in 2020−22 was decomposed into numerous causes of death at specific ages. The causes depicted here are the leading six contributors to this life expectancy gap. *Abbreviations*. NH (Non-Hispanic); AIAN (American Indian or Alaska Native).

The age distribution of the top six contributors to racial/ethnic life expectancy gaps in 2020−22 is depicted in Fig 2 for males and Fig 3 for females. Among both males and females, these causes of death made only modest contributions to longevity gaps prior to the age of 15. In their late teens and 20s, fatalities due to alcohol-related motor vehicle crashes increased substantially for NH AIAN, particularly among males. Among both males (Fig 2) and females (Fig 3) in their late 20s through their early 50s, mortality from alcohol abuse and poisoning and especially from alcoholic liver disease made large contributions to racial/ethnic longevity gaps. To illustrate, alcoholic liver disease contributed over 0.30 years to the racial/ethnic longevity gap at age 35−39 alone, regardless of sex. While COVID-19 made the largest contributions to life expectancy gaps, it did so at relatively younger ages for males, who experienced maximum effects in their 40s, than for females, who experienced maximum effects in their 60s. Other notable contributors among males were non-alcoholic isoning (e.g., accidental drug overdose), which peaked in their late 30s, and other external causes or unspecified accidents, which peaked during their early 40s. Among females, other important contributors included alcohol-related unspecified liver diseases, which had the largest impact between ages 30 and 54, and diabetes mellitus, which gradually increased in magnitude from mid-to-late life.

Table 2 presents the contribution of select causes of death to life expectancy changes within each sex and racial/ethnic group, expressed as years lost (negative change) from 2018−19–2020−22. During the pandemic, life expectancy at birth declined by 7.33 years for NH AIAN males and 6.76 years for AIAN females; by comparison, life expectancy declined by 2.11 and 1.72 years among NH White males and females, respectively. COVID-19 was the most substantial contributor to these declines in life expectancy, accounting for at least 50% of longevity change in each race/sex group. Among NH AIAN males, other important contributors to the 7.33-year loss in life expectancy included non-alcohol (i.e., other drug) poisoning (−0.81 years), alcoholic liver disease (−0.57 years), and alcohol abuse and poisoning (−0.29 years). Among NH AIAN females, alcoholic liver disease (−0.49 years), non-alcohol poisoning (−0.35 years), and alcohol-related liver cirrhosis (−0.33) made notable contributions to the 6.76-year decline in longevity. For both NH White males and females, diseases of the heart and non-alcohol poisoning were important contributors to the roughly two-year loss of life expectancy experienced by each group.

**Table 2:** Contribution of select causes of death to change in life expectancy between peak pandemic and pre-pandemic time periods in the Four Corners States, by race/ethnicity and sex.

Causes of Death^1^	NH AIAN		NH White	
**Male**	**Female**	**Male**	**Female**
2020-2022 (peak pandemic)	61.11	70.69	76.08	80.93
2018-2019 (pre-pandemic)	68.44	77.45	78.19	82.65
**Life expectancy change between peak and pre-pandemic time periods**	**−7.33**	**−6.76**	**−2.11**	**−1.72**
COVID-19	−4.05	−4.21	−1.12	−0.86
Poisoning (not alcohol)	−0.81	−0.35	−0.27	−0.11
Alcoholic liver disease	−0.57	−0.49	−0.06	−0.06
Diseases of heart	−0.22	−0.05	−0.18	−0.16
Alcohol-related liver cirrhosis	−0.17	−0.33	−0.02	−0.02
Alcohol abuse and poisoning	−0.29	−0.17	−0.04	−0.02
All other causes	−1.21	−1.16	−0.42	−0.50

*Note.* 1) Among the 39 leading causes of death and ARDI we examined, select causes of death were included in this table if they contributed at least 0.20 years to changes in life expectancy for at least one race/sex group that occurred after the onset of COVID-19. Causes of death are listed in order of total contribution to changes in life expectancy. A residual category (all other causes) accounts for the total contribution of all other causes of death that did not meet our inclusion criteria. Negative contributions indicate declines in life expectancies between these two time periods.

*Abbreviations*. NH (Non-Hispanic); AIAN (American Indian or Alaska Native); ARDI (Alcohol-Related Disease Impact)

## Discussion

Using restricted-use NCHS data from 2018−2022, our study investigated trends and racial disparities in life expectancy in the FCS during the COVID-19 pandemic. We assessed the relative contributions of COVID-19 and other causes of death to life expectancy decline within racial groups and to disparities between racial groups. Our results were consistent with findings from previous research indicating high mortality rates and declines in life expectancy due to COVID-19 and alcohol-related causes of deaths among indigenous people in the FCS [1–3,5,6,31].

The COVID-19 pandemic had a detrimental impact on life expectancy and exacerbated pre-existing longevity disparities between NH AIAN and NH Whites in the FCS. Among NH AIAN in the FCS, longevity decreased by 7.33 years for males and 6.76 years for females between 2018−19 and 2020−22. Goldman and Andrasfay found an average life expectancy decrease of 6.4 years for the overall US population of NH AIAN between 2019 and 2021 [5]. Using that same metric (see our 2019 and 2021 estimates in Fig 1), our findings showed an even greater reduction in longevity (>8 years) among NH AIAN males and females in the FCS. These results support prior studies which argue that tribes within the FCS were especially vulnerable to the COVID-19 pandemic due to various structural inequalities [2,3,20,21].

Alcohol use disorder, which is associated with both acute and chronic causes of death and has higher prevalence among NH AIAN than NH Whites [28,32–35]. Alcohol use disorder contributes to a stark mortality gap, with death rates from excessive alcohol use reaching 145.3 per 100,000 in the AIAN population, compared to 63.8 in the NH White population [31]. Our results indicate that alcohol-related deaths contributed substantially to the racial gap in life expectancy in the FCS. Furthermore, our analysis advances this literature by quantifying the age-specific contributions of alcohol-related causes of death to longevity disparities between NH AIAN and NH White populations in the FCS.

Relative to other causes of death, COVID-19 was the single largest contributor to the reduction in life expectancy for both NH AIAN and NH Whites in the FCS. Longevity among NH AIAN males and females also decreased *during the pandemic* due to heart disease, non-alcohol poisoning, alcoholic liver disease, and alcohol abuse and poisoning. This suggests that alcohol and other substances may have been used as coping mechanisms during this time of distress and social isolation among NH AIAN in the FCS. Among NH White males and females, life expectancy declined during the pandemic due to some causes of death other than COVID-19 but to a much lesser degree.

With respect to the widening longevity gap between NH AIAN and NH Whites in the FCS, COVID-19 was the single largest contributor (>3 years for both males and females) during the pandemic. In addition, alcoholic and alcohol-related liver diseases, alcohol abuse and poisoning, non-alcohol poisoning, and alcohol-related suicide all made larger contributions to longevity gaps between NH AIAN and NH Whites during the pandemic than in the previous period (i.e., 2018−19), regardless of sex. Other important contributors to life expectancy disparities such as alcohol-related homicide, alcohol-related vehicle crashes, and diabetes either declined slightly or differed between sexes in their impact during the pandemic but continued to make notable contributions. During the pandemic, substance use, suicidality, and mental illness symptoms increased due to stress, isolation, and trauma—especially among AIAN individuals [36–38]. The negative mental health effects of trauma and loss of cultural and community roots historically experienced by AIAN individuals created vulnerability to the adverse effects of the COVID-19 pandemic, unfortunately including sharp increases in mortality [38–40].

### Public health implications

Despite inherent difficulties in overcoming the social and economic disadvantages faced by AIAN that contribute to reduced health and longevity, research suggests that they can be addressed with targeted interventions. Structural inequalities such as poverty, food insecurity, incomplete plumbing, overcrowded housing, inadequate access to transportation, and barriers to healthcare services (e.g., lower rates of health insurance coverage) all contribute to insufficient preventive care and increased risk for morbidity, including severe cases of COVID-19 [2,3,13,14,16–21,41–43]. Discrimination against indigenous groups and historical events have contributed to mistrust of the health care system and vaccination hesitancy, also placing AIAN individuals at heightened risk for COVID-19 and its complications [17,18]. Cultural sensitivity and improved representation of indigenous people among public health and medical professionals may help overcome distrust and other healthcare barriers, contributing to improved vaccine uptake and better general preventive care.

Drug and alcohol use often coincide with mental illness and trauma [44,45]. Native American adolescents and young adults have high rates of mental illness, particularly as they relate to substance and alcohol use disorders [46,47]. Mental illness, substance use disorders, and alcohol and drug use are all risk factors for accidental deaths (e.g., fatal motor vehicle accidents), homicide, and suicide [48,49]. The mortality age patterns we found suggest that early interventions to support mental health and prevent alcohol use are crucial within the AIAN population. Culturally informed and community-based interventions are crucial to reducing deaths associated with underlying mental illness such as alcohol and drug overdose, suicide, and homicide among indigenous populations [40,50].

### Strengths and limitations

Restricted-use NCHS data helped us achieve accurate numerator counts, especially in rural counties where public mortality data tend to be censored due to low numbers. In addition, our use of decomposition methods enabled us to assess how numerous causes of death at different ages contributed to life expectancy changes and gaps during the height of the COVID-19 pandemic. Although we explained most of the life expectancy gaps and changes over this period of observation, 15–30% of these gaps and changes were unaccounted for by the causes of death examined in our analyses. While restricted-use NCHS data provide the most accurate mortality estimates available, they are not linked to economic data or health history information, limiting the ability of analysts to account for these factors. Finally, racial misclassification on death records is common among NH AIAN individuals in the US and may be more prevalent in urban areas and populations with a low percentage of NH AIAN people [51,52]. However, prior research has shown that the Southwest region, which encompasses the FCS, has a much lower misclassification rate than other US regions [51]. Therefore, because our study population lies within the Southwest and is predominantly rural with large NH AIAN majorities in some areas, we expect misclassification bias to be minimal.

## Conclusions

In the FCS, life expectancy declined among both NH AIAN and NH Whites during the COVID-19 pandemic. However, indigenous people were disproportionately impacted by the pandemic, which exacerbated pre-existing longevity disparities. Current public health initiatives and policies are not adequately addressing health and mortality disparities among indigenous groups in the US [41]. To improve NH AIAN well-being and prevent losses to future health crises such as infectious disease outbreaks in the FCS, access to preventive care and public health responses need to be improved in this population. Creative and concerted efforts from all parties can bolster extant programs and catalyze the development of novel interventions that are designed to address difficult health challenges faced by NH AIAN in the FCS, helping to alleviate suffering and premature mortality in this group of Native Americans.

## Supporting information

S1 Table List of ICD10 codes by the 39 leading causes of death and alcohol-related disease impact classification. Sources: CDC, National Center for Health Statistics. Instruction Manual ICD-10 Cause-of-Death Lists for Tabulating Mortality Statistics. 2009; CDC. Alcohol Related Disease Impact (ARDI) Application Website. 2024. Available: www.cdc.gov/ARDI. (PDF)
