# Supplementary material for: The impact of COVID-19 on longevity trends and disparities among Native Americans and Whites in the Four Corners States
Source: PLoS One. 2026 May 18;21(5):e0347924. doi: 10.1371/journal.pone.0347924 (PMC13183185; doi:10.1371/journal.pone.0347924)
Supplement: S1 Table — (PDF) [file pone.0347924.s001.pdf]

S1 Table. List of ICD10 codes by the 39 leading causes of death and alcohol-related disease impact classification

The 39 leading causes of death	Alcohol-related disease impact (ARDI) classification	ICD10
Alzheimer's disease		G300 G301 G308 G309
Assault (homicide)	ARDI_Homicide	X85 X86 X87 X88 X89 X90 X91 X92 X93 X94 X95 X96 X97 X98 X99 Y00 Y01 Y02 Y03 Y04 Y05 Y060 Y061 Y068 Y069 Y070 Y071 Y072 Y073 Y078 Y079 Y08 Y09 Y871
Assault (homicide)		U012 U014 U018 U02
Atherosclerosis		I700 I701 I702 I708 I709
COVID-19		U071
Cerebrovascular diseases		I600 I601 I602 I603 I604 I605 I606 I607 I608 I609 I610 I611 I612 I613 I614 I615 I616 I618 I619 I620 I621 I629 I630 I631 I632 I633 I634 I635 I636 I638 I639 I64 I670 I671 I672 I673 I674 I675 I676 I677 I678 I679 I690 I691 I692 I693 I694 I698
Certain conditions originating in the perinatal period		P000 P001 P002 P003 P004 P005 P006 P007 P008 P009 P010 P011 P012 P013 P014 P015 P016 P017 P018 P019 P020 P021 P022 P023 P024 P025 P026 P027 P028 P029 P030 P031 P032 P033 P034 P035 P036 P038 P039 P040 P041 P042 P043 P044 P046 P048 P049 P050 P051 P052 P059 P070 P071 P072 P073 P082 P100 P101 P102 P103 P109 P110 P111 P112 P113 P115 P119 P120 P122 P128 P129 P130 P131 P139 P140 P149 P150 P151 P154 P158 P159 P200 P201 P209 P210 P219 P220 P221 P228 P229 P230 P231 P232 P233 P234 P235 P236 P238 P239 P240 P241 P242 P243 P248 P249 P250 P251 P252 P253 P258 P260 P261 P268 P269 P270 P271 P278 P279 P280 P281 P282 P283 P284 P285 P288 P289 P290 P291 P292 P293 P294 P298 P299 P350 P351 P352 P353 P358 P359 P360 P361 P362 P363 P364 P365 P368 P369 P370 P371 P372 P373 P374 P375 P378 P38 P391 P392 P393 P394 P398 P399 P500 P501 P502 P504 P508 P509 P510 P518 P519 P520 P521 P522 P523 P524 P525 P526 P528 P529 P53 P542 P543 P544 P545 P546 P548 P549 P550 P551 P558 P559 P560 P570 P579 P580 P582 P584 P588 P591 P592 P598 P599 P60 P610 P611 P612 P613 P614 P615 P616 P618 P619 P700 P701 P702 P704 P708 P721 P740 P741 P743 P749 P760 P761 P768 P769 P77 P780 P781 P783 P788 P789 P800 P808 P809 P810 P818 P819 P832 P833 P835 P838 P839 P90 P910 P911 P912 P914 P915 P916 P918 P919 P920 P921 P923 P924 P928 P929 P940 P941 P942 P948 P949 P960 P961 P962 P964 P965 P968 P969
Chronic liver disease and cirrhosis	ARDI_Alcoholic liver disease	K700 K701 K702 K703 K704 K709
Chronic liver disease and cirrhosis	ARDI_Liver cirrhosis, unspecified	K740 K741 K742 K746
Chronic liver disease and cirrhosis		K730 K732 K738 K739 K743 K744 K745
Chronic lower respiratory diseases		J40 J410 J411 J418 J42 J430 J431 J432 J438 J439 J440 J441 J448 J449 J450 J451 J458 J459 J46 J47

The 39 leading causes of death	Alcohol-related disease impact (ARDI) classification	ICD10
Congenital malformations, deformations and chromosomal abnormalities.		Q000 Q001 Q002 Q010 Q011 Q012 Q018 Q019 Q02 Q030 Q031 Q038 Q039 Q040 Q041 Q042 Q043 Q044 Q045 Q046 Q048 Q049 Q050 Q051 Q052 Q054 Q055 Q056 Q057 Q058 Q059 Q060 Q061 Q062 Q064 Q068 Q069 Q070 Q078 Q079 Q103 Q111 Q112 Q120 Q130 Q131 Q134 Q138 Q148 Q150 Q158 Q159 Q161 Q171 Q172 Q180 Q181 Q182 Q184 Q185 Q188 Q189 Q200 Q201 Q202 Q203 Q204 Q205 Q206 Q208 Q209 Q210 Q211 Q212 Q213 Q214 Q218 Q219 Q220 Q221 Q222 Q223 Q224 Q225 Q226 Q228 Q229 Q230 Q231 Q232 Q233 Q234 Q238 Q239 Q240 Q241 Q242 Q243 Q244 Q245 Q246 Q248 Q249 Q250 Q251 Q252 Q253 Q254 Q255 Q256 Q257 Q258 Q259 Q261 Q262 Q263 Q264 Q265 Q266 Q268 Q269 Q270 Q271 Q272 Q273 Q274 Q278 Q279 Q280 Q281 Q282 Q283 Q288 Q289 Q300 Q301 Q308 Q309 Q310 Q311 Q312 Q313 Q315 Q318 Q319 Q320 Q321 Q322 Q323 Q324 Q330 Q332 Q333 Q334 Q336 Q338 Q339 Q340 Q341 Q348 Q349 Q351 Q359 Q361 Q369 Q378 Q379 Q382 Q383 Q385 Q386 Q387 Q388 Q390 Q391 Q392 Q393 Q394 Q395 Q396 Q398 Q399 Q400 Q401 Q402 Q403 Q408 Q409 Q410 Q411 Q412 Q418 Q419 Q421 Q422 Q423 Q428 Q429 Q430 Q431 Q432 Q433 Q434 Q435 Q437 Q438 Q439 Q440 Q441 Q442 Q443 Q444 Q445 Q446 Q447 Q450 Q451 Q453 Q458 Q459 Q502 Q505 Q506 Q510 Q512 Q513 Q514 Q518 Q519 Q520 Q522 Q526 Q527 Q529 Q532 Q539 Q549 Q554 Q555 Q558 Q559 Q560 Q564 Q600 Q601 Q602 Q603 Q604 Q605 Q606 Q610 Q611 Q612 Q613 Q614 Q615 Q618 Q619 Q620 Q621 Q622 Q623 Q624 Q625 Q626 Q627 Q628 Q630 Q631 Q632 Q633 Q638 Q639 Q641 Q642 Q643 Q644 Q645 Q646 Q647 Q648 Q649 Q651 Q652 Q658 Q659 Q660 Q661 Q665 Q668 Q670 Q672 Q673 Q675 Q676 Q677 Q678 Q688 Q699 Q702 Q703 Q709 Q713 Q718 Q719 Q724 Q728 Q729 Q730 Q731 Q738 Q740 Q741 Q742 Q743 Q748 Q749 Q750 Q751 Q752 Q753 Q754 Q758 Q759 Q760 Q761 Q762 Q764 Q766 Q767 Q768 Q769 Q770 Q771 Q772 Q773 Q774 Q775 Q776 Q777 Q778 Q779 Q780 Q781 Q782 Q783 Q784 Q785 Q786 Q788 Q789
Diabetes mellitus		E100 E101 E102 E103 E104 E105 E106 E107 E108 E109 E110 E111 E112 E113 E114 E115 E116 E117 E118 E119 E120 E121 E122 E124 E125 E126 E127 E129 E130 E131 E132 E134 E135 E136 E137 E138 E139 E140 E141 E142 E143 E144 E145 E146 E147 E148 E149
Essential (primary) hypertension and hypertensive renal disease		I10 I120 I129 I150 I159
Human immunodeficiency virus (HIV) disease		B200 B201 B202 B203 B204 B205 B206 B207 B208 B209 B210 B211 B212 B213 B217 B218 B219 B220 B222 B227 B231 B232 B238 B24
Hypertensive heart disease with or without renal disease		I110 I119 I130 I131 I132 I139

The 39 leading causes of death	Alcohol-related disease impact (ARDI) classification	ICD10
Influenza and pneumonia		J09 J100 J101 J108 J110 J111 J118 J120 J121 J122 J123 J128 J129 J13 J14 J150 J151 J152 J153 J154 J155 J156 J157 J158 J159 J160 J168 J180 J181 J182 J188 J189
Intentional self-harm (suicide)	ARDI_Alcohol abuse and poisoning	X65
Intentional self-harm (suicide)	ARDI_Suicide	X60 X61 X62 X63 X64 X66 X67 X68 X69 X70 X71 X72 X73 X74 X75 X76 X77 X78 X79 X80 X81 X82 X83 X84
Intentional self-harm (suicide)		Y870
Ischemic heart diseases		I200 I201 I208 I209 I210 I211 I212 I213 I214 I219 I220 I221 I228 I229 I241 I248 I249 I250 I251 I252 I253 I254 I255 I256 I258 I259
Leukemia		C910 C911 C912 C913 C914 C915 C917 C919 C920 C921 C922 C923 C924 C925 C927 C929 C930 C931 C937 C939 C940 C941 C942 C943 C944 C945 C947 C950 C951 C952 C957 C959
Malignant neoplasm of breast		C500 C501 C502 C503 C504 C505 C506 C508 C509
Malignant neoplasm of pancreas		C250 C251 C252 C253 C254 C257 C258 C259
Malignant neoplasm of prostate		C61
Malignant neoplasm of stomach		C160 C161 C162 C163 C164 C165 C166 C168 C169
Malignant neoplasms of cervix uteri, corpus uteri and ovary		C530 C531 C538 C539 C540 C541 C542 C543 C548 C549 C55 C56
Malignant neoplasms of colon, rectum and anus		C180 C181 C182 C183 C184 C185 C186 C187 C188 C189 C19 C20 C210 C211 C212 C218
Malignant neoplasms of trachea, bronchus and lung		C33 C340 C341 C342 C343 C348 C349
Malignant neoplasms of urinary tract		C64 C65 C66 C670 C671 C672 C673 C674 C675 C676 C677 C678 C679 C680 C681 C688 C689

The 39 leading causes of death	Alcohol-related disease impact (ARDI) classification	ICD10
Motor vehicle accidents	ARDI_Motor vehicle traffic crashes	V021 V029 V031 V039 V041 V049 V092 V124 V125 V129 V133 V134 V135 V139 V143 V144 V145 V149 V194 V195 V196 V204 V205 V209 V214 V215 V219 V223 V224 V225 V229 V233 V234 V235 V239 V243 V244 V245 V249 V254 V255 V259 V263 V264 V265 V269 V273 V274 V275 V279 V283 V284 V285 V289 V294 V295 V296 V298 V299 V305 V306 V309 V315 V324 V325 V329 V334 V335 V336 V339 V344 V345 V346 V349 V355 V359 V365 V374 V375 V376 V379 V384 V385 V386 V389 V394 V395 V396 V398 V399 V404 V405 V406 V407 V409 V414 V415 V416 V419 V424 V425 V426 V427 V429 V434 V435 V436 V437 V439 V444 V445 V446 V447 V449 V454 V455 V456 V457 V459 V464 V465 V466 V467 V469 V474 V475 V476 V477 V479 V484 V485 V486 V487 V489 V494 V495 V496 V498 V499 V504 V505 V506 V507 V509 V515 V516 V519 V525 V526 V529 V534 V535 V536 V537 V539 V544 V545 V546 V547 V549 V554 V555 V556 V559 V564 V565 V566 V569 V574 V575 V576 V577 V579 V584 V585 V586 V587 V589 V594 V595 V596 V598 V599 V604 V605 V606 V607 V609 V619 V625 V629 V634 V635 V636 V637 V639 V644 V645 V646 V647 V649 V654 V655 V656 V659 V665 V666 V669 V674 V675 V676 V677 V679 V684 V685 V686 V687 V689 V694 V695 V696 V698 V699 V706 V724 V726 V729 V735 V736 V739 V745 V746 V749 V755 V756 V766 V769 V775 V776 V779 V784 V785 V786 V789 V794 V795 V796 V798 V799 V803 V804 V805 V811 V830 V831 V832 V833 V840 V841 V842 V843 V850 V851 V852 V853 V860 V861 V862 V863 V870 V871 V872 V873 V892
Motor vehicle accidents		V020 V030 V040 V090 V120 V130 V131 V132 V140 V141 V142 V190 V191 V192 V200 V201 V202 V210 V220 V221 V222 V230 V231 V232 V240 V242 V250 V251 V252 V260 V270 V271 V272 V280 V281 V282 V290 V291 V292 V293 V301 V320 V370 V380 V383 V392 V393 V400 V401 V403 V420 V423 V430 V431 V432 V433 V440 V441 V443 V450 V451 V453 V460 V463 V470 V471 V472 V473 V480 V481 V482 V483 V490 V491 V492 V493 V500 V501 V502 V530 V531 V533 V540 V541 V543 V550 V551 V553 V570 V571 V572 V573 V580 V581 V582 V583 V590 V591 V592 V593 V601 V602 V603 V640 V641 V642 V643 V650 V651 V653 V660 V670 V671 V672 V673 V680 V681 V682 V683 V690 V691 V692 V693 V770 V771 V773 V780 V781 V782 V783 V790 V791 V792 V793 V810 V834 V835 V836 V837 V839 V844 V845 V846 V847 V849 V854 V855 V856 V857 V859 V864 V865 V866 V867 V869 V874 V875 V876 V877 V878 V880 V881 V882 V883 V884 V885 V886 V887 V888 V890

The 39 leading causes of death	Alcohol-related disease impact (ARDI) classification	ICD10
Nephritis, nephrotic syndrome and nephrosis		N000 N001 N005 N007 N008 N009 N012 N017 N018 N019 N022 N028 N029 N031 N032 N035 N037 N038 N039 N040 N041 N045 N047 N048 N049 N050 N051 N052 N053 N055 N056 N057 N058 N059 N071 N079 N170 N171 N172 N178 N179 N180 N181 N182 N183 N184 N185 N188 N189 N19 N250 N251 N258 N259 N26 N271 N279
Non-Hodgkin's lymphoma		C820 C821 C822 C827 C829 C830 C831 C832 C833 C834 C835 C836 C837 C838 C839 C840 C841 C842 C843 C844 C845 C850 C851 C857 C859
Other diseases of circulatory system		I710 I711 I712 I713 I714 I715 I716 I718 I719 I720 I721 I722 I723 I724 I725 I728 I729 I730 I731 I738 I739 I740 I741 I742 I743 I744 I745 I748 I749 I770 I771 I772 I773 I774 I775 I776 I778 I779 I780 I781 I788 I789
Other diseases of heart		I00 I010 I011 I012 I018 I019 I029 I050 I051 I052 I058 I059 I060 I061 I062 I068 I069 I070 I071 I072 I078 I079 I080 I081 I082 I083 I088 I089 I090 I091 I092 I098 I099 I260 I269 I270 I271 I272 I278 I279 I280 I281 I288 I289 I300 I301 I308 I309 I310 I311 I312 I313 I318 I319 I330 I339 I340 I341 I342 I348 I349 I350 I351 I352 I358 I359 I360 I361 I362 I368 I369 I370 I371 I372 I378 I379 I38 I400 I401 I408 I409 I420 I421 I422 I423 I424 I425 I426 I427 I428 I429 I440 I441 I442 I443 I444 I446 I447 I450 I451 I452 I453 I454 I455 I456 I458 I459 I460 I461 I469 I470 I471 I472 I479 I48 I490 I491 I493 I494 I495 I498 I499 I500 I501 I509 I510 I511 I512 I513 I514 I515 I516 I517 I518 I519

The 39 leading causes of death	Alcohol-related disease impact (ARDI) classification	ICD10
Other malignant neoplasms		C000 C001 C004 C005 C006 C008 C009 C01 C020 C021 C022 C023 C024 C028 C029 C030 C031 C039 C040 C041 C048 C049 C050 C051 C052 C058 C059 C060 C061 C062 C068 C069 C07 C080 C081 C089 C090 C091 C098 C099 C100 C101 C102 C103 C104 C108 C109 C110 C111 C112 C113 C118 C119 C12 C130 C131 C132 C138 C139 C140 C142 C148 C150 C151 C152 C153 C154 C155 C158 C159 C170 C171 C172 C173 C178 C179 C220 C221 C222 C223 C224 C227 C229 C23 C240 C241 C248 C249 C260 C261 C268 C269 C300 C301 C310 C311 C312 C313 C318 C319 C320 C321 C322 C323 C328 C329 C37 C380 C381 C382 C383 C384 C388 C390 C398 C399 C400 C401 C402 C403 C408 C409 C410 C411 C412 C413 C414 C418 C419 C430 C431 C432 C433 C434 C435 C436 C437 C438 C439 C440 C441 C442 C443 C444 C445 C446 C447 C448 C449 C450 C451 C452 C457 C459 C460 C461 C462 C463 C467 C468 C469 C470 C471 C472 C473 C474 C475 C476 C478 C479 C480 C481 C482 C488 C490 C491 C492 C493 C494 C495 C496 C498 C499 C510 C511 C512 C518 C519 C52 C570 C571 C572 C573 C574 C577 C578 C579 C58 C600 C601 C602 C608 C609 C620 C621 C629 C630 C631 C632 C637 C638 C639 C690 C691 C692 C693 C694 C695 C696 C698 C699 C700 C701 C709 C710 C711 C712 C713 C714 C715 C716 C717 C718 C719 C720 C721 C722 C723 C724 C725 C728 C729 C73 C740 C741 C749 C750 C751 C752 C753 C754 C755 C758 C759 C760 C761 C762 C763 C764 C765 C767 C768 C770 C771 C772 C773 C774 C775 C778 C779 C780 C781 C782 C783 C784 C785 C786 C787 C788 C790 C791 C792 C793 C794 C795 C796 C797 C798 C80 C810 C811 C812 C813 C817 C819 C880 C881 C882 C883 C887 C889 C900 C901 C902 C960 C961 C962 C967 C969 C97
Peptic ulcer		K250 K251 K252 K253 K254 K255 K256 K257 K259 K260 K261 K262 K263 K264 K265 K266 K267 K269 K270 K271 K272 K273 K274 K275 K276 K277 K279 K280 K281 K283 K284 K285 K286 K287 K289

The 39 leading causes of death	Alcohol-related disease impact (ARDI) classification	ICD10
Pregnancy, childbirth and the puerperium		O000 O001 O002 O008 O009 O019 O020 O021 O029 O031 O032 O033 O035 O036 O037 O038 O039 O040 O045 O047 O048 O049 O051 O054 O056 O058 O059 O060 O063 O065 O066 O067 O068 O069 O073 O074 O075 O076 O077 O078 O100 O101 O102 O103 O104 O109 O11 O120 O121 O13 O140 O141 O142 O149 O150 O152 O159 O16 O200 O208 O209 O210 O211 O222 O223 O225 O228 O229 O230 O234 O235 O240 O241 O243 O244 O249 O25 O263 O264 O265 O266 O268 O269 O291 O292 O300 O301 O321 O339 O340 O341 O343 O345 O348 O360 O363 O364 O365 O368 O40 O411 O429 O431 O432 O438 O441 O450 O459 O460 O468 O469 O60 O620 O621 O622 O623 O628 O639 O644 O669 O670 O678 O679 O680 O689 O690 O698 O701 O709 O710 O711 O713 O714 O715 O716 O717 O720 O721 O722 O723 O730 O731 O742 O743 O746 O748 O749 O750 O751 O753 O754 O755 O757 O758 O759 O85 O860 O864 O868 O870 O871 O873 O878 O879 O880 O881 O882 O883 O888 O898 O900 O901 O903 O904 O905 O908 O909 O911 O912 O95 O96 O960 O961 O969 O97 O970 O971 O979 O980 O981 O982 O984 O985 O986 O987 O988 O990 O991 O992 O993 O994 O995 O996 O997 O998
Sudden infant death syndrome.		R95
Symptoms, signs and abnormal clinical and laboratory findings, not elsewhere classified (excluding Sudden infant death syndrome)		R000 R001 R002 R008 R011 R02 R030 R040 R041 R042 R048 R049 R05 R060 R061 R062 R063 R064 R065 R066 R067 R068 R070 R072 R073 R074 R090 R091 R092 R093 R098 R100 R101 R102 R103 R104 R11 R12 R13 R14 R160 R161 R162 R17 R18 R190 R194 R198 R200 R203 R208 R21 R220 R221 R222 R223 R224 R229 R230 R233 R234 R238 R251 R252 R258 R260 R263 R268 R270 R278 R290 R291 R292 R298 R300 R31 R32 R33 R34 R35 R391 R398 R400 R401 R402 R410 R413 R418 R42 R430 R432 R441 R448 R450 R451 R453 R456 R458 R460 R461 R468 R470 R471 R478 R482 R488 R490 R508 R509 R51 R520 R522 R529 R53 R54 R55 R560 R568 R570 R571 R578 R579 R58 R590 R591 R599 R600 R601 R609 R628 R629 R630 R631 R632 R633 R634 R635 R636 R638 R64 R680 R688 R701 R71 R72 R730 R739 R740 R748 R749 R75 R768 R778 R779 R780 R781 R782 R783 R784 R788 R798 R80 R823 R825 R849 R859 R897 R900 R908 R91 R930 R932 R933 R934 R935 R938 R940 R942 R943 R944 R945 R946 R960 R961 R98 R99
Syphilis		A502 A503 A504 A505 A506 A509 A513 A514 A515 A520 A521 A523 A527 A528 A529 A530 A539
Tuberculosis		A162 A163 A164 A165 A167 A168 A169 A170 A178 A179 A180 A181 A182 A183 A184 A185 A187 A188 A190 A191 A192 A198 A199
All other and unspecified accidents and adverse effects	ARDI_Alcohol abuse and poisoning	X45

The 39 leading causes of death	Alcohol-related disease impact (ARDI) classification	ICD10
All other and unspecified accidents and adverse effects	ARDI_Poisoning (not alcohol)	X40 X41 X42 X43 X44 X46 X47 X48 X49
All other and unspecified accidents and adverse effects		V010 V011 V019 V050 V051 V059 V060 V061 V069 V091 V093 V099 V100 V102 V104 V109 V110 V112 V114 V119 V150 V151 V152 V154 V155 V159 V162 V164 V169 V170 V172 V174 V175 V179 V180 V181 V182 V183 V184 V185 V189 V193 V198 V199 V800 V801 V807 V808 V809 V812 V813 V814 V815 V816 V817 V818 V819 V822 V824 V825 V826 V827 V829 V879 V889 V891 V893 V899 V900 V901 V902 V903 V904 V905 V906 V907 V908 V909 V910 V911 V912 V913 V914 V915 V916 V917 V918 V919 V920 V921 V922 V923 V924 V925 V926 V927 V928 V929 V930 V931 V932 V933 V934 V935 V936 V938 V939 V940 V941 V942 V943 V944 V945 V946 V947 V948 V949 V950 V951 V952 V953 V958 V959 V960 V961 V962 V968 V969 V970 V971 V972 V973 V978 V98 V99 W00 W01 W02 W03 W04 W05 W06 W07 W08 W09 W10 W11 W12 W13 W14 W15 W16 W17 W18 W19 W20 W21 W22 W23 W24 W25 W26 W27 W28 W29 W30 W31 W32 W33 W34 W35 W36 W37 W38 W39 W40 W41 W42 W44 W45 W46 W49 W50 W51 W52 W53 W54 W55 W56 W57 W58 W59 W60 W64 W65 W66 W67 W68 W69 W70 W73 W74 W75 W76 W77 W78 W79 W80 W81 W83 W84 W85 W86 W87 W89 W91 W92 W93 W94 X00 X01 X02 X03 X04 X05 X06 X08 X09 X10 X11 X12 X13 X14 X15 X16 X17 X18 X19 X20 X21 X22 X23 X24 X25 X26 X29 X30 X31 X32 X33 X34 X349 X36 X37 X38 X39 X50 X51 X52 X53 X54 X57 X58 X59 X590 X599 Y400 Y401 Y402 Y403 Y404 Y405 Y407 Y408 Y409 Y410 Y411 Y412 Y414 Y415 Y418 Y419 Y420 Y421 Y422 Y423 Y424 Y425 Y426 Y427 Y428 Y429 Y430 Y431 Y433 Y434 Y435 Y436 Y439 Y440 Y441 Y442 Y443 Y445 Y446 Y447 Y449 Y450 Y451 Y452 Y453 Y454 Y455 Y458 Y459 Y461 Y462 Y464 Y465 Y466 Y467 Y468 Y470 Y471 Y478 Y479 Y480 Y481 Y482 Y483 Y484 Y485 Y490 Y492 Y493 Y494 Y495 Y496 Y497 Y498 Y499 Y501 Y509 Y513 Y515 Y517 Y518 Y519 Y520 Y521 Y522 Y523 Y524 Y525 Y526 Y527 Y528 Y529 Y531 Y533 Y535 Y536 Y540 Y542 Y543 Y544 Y545 Y546 Y548 Y549
All other diseases (Residual)	ARDI_Alcohol abuse and poisoning	F100 F101
All other diseases (Residual)	ARDI_Liver cirrhosis, unspecified	K760 K767 K769

The 39 leading causes of death	Alcohol-related disease impact (ARDI) classification	ICD10
All other diseases (Residual)		A000 A009 A010 A014 A020 A021 A022 A029 A030 A033 A038 A039 A040 A041 A042 A043 A044 A045 A046 A047 A048 A049 A051 A052 A053 A059 A060 A062 A064 A065 A066 A067 A068 A069 A071 A072 A073 A080 A081 A082 A083 A084 A085 A09 A090 A099 A202 A207 A209 A212 A217 A219 A230 A239 A241 A242 A244 A251 A259 A267 A269 A270 A279 A280 A281 A282 A288 A300 A305 A309 A310 A311 A318 A319 A321 A327 A328 A329 A35 A369 A370 A371 A378 A379 A38 A390 A391 A392 A394 A395 A398 A399 A400 A401 A402 A403 A408 A409 A410 A411 A412 A413 A414 A415 A418 A419 A420 A421 A422 A427 A428 A429 A430 A431 A438 A439 A440 A449 A46 A480 A481 A482 A483 A488 A490 A491 A492 A493 A498 A499 A540 A542 A544 A545 A548 A549 A55 A560 A57 A58 A599 A600 A601 A630 A64 A690 A691 A692 A70 A749 A752 A759 A770 A773 A778 A779 A78 A791 A798 A799 A810 A811 A812 A818 A819 A829 A830 A832 A833 A834 A835 A838 A839 A848 A849 A850 A851 A858 A86 A870 A872 A878 A879 A888 A89 A90 A91 A920 A923 A924 A928 A938 A94 A959 A962 A984 A985 A99 B000 B001 B002 B003 B004 B005 B007 B008 B009 B010 B011 B012 B018 B019 B020 B021 B022 B023 B027 B028 B029 B04 B050 B051 B052 B059 B060 B068 B069 B07 B083 B084 B085 B09 B150 B159 B160 B162 B169 B171 B172 B178 B179 B180 B181 B182 B188 B189 B190 B199 B250 B251 B252 B258 B259 B261 B269 B270 B271 B278 B279 B302 B308 B332 B333 B334 B338 B340 B341 B342 B343 B344 B348 B349 B350 B351 B352 B353 B354 B356 B358 B359 B360 B362 B368 B369 B370 B371 B372 B373 B374 B375 B376 B377 B378 B379 B380 B381 B382 B383 B384 B387 B388 B389 B390 B391 B392 B393 B394 B399 B400 B401 B402 B403 B407 B408 B409 B419 B420 B421 B427 B428 B429 B431 B438 B439 B440 B441 B447 B448 B449 B450 B451 B452 B453 B457 B458 B459 B460 B461 B462 B463 B464 B465 B468 B469 B471 B479 B481 B482 B483 B484 B487 B488 B49 B500 B508
All other external causes	ARDI_Poisoning (not alcohol)	Y10 Y11 Y12 Y13 Y14 Y16 Y17 Y18 Y19
All other external causes		Y15 Y20 Y21 Y22 Y23 Y24 Y25 Y26 Y27 Y28 Y29 Y30 Y31 Y32 Y33 Y34 Y350 Y351 Y352 Y353 Y355 Y356 Y357 Y362 Y364 Y367 Y368 Y369 Y872 Y890 Y891 Y899
